# Unique functions of CHK1 and WEE1 underlie synergistic anti-tumor activity upon pharmacologic inhibition

**DOI:** 10.1186/1475-2867-12-45

**Published:** 2012-11-13

**Authors:** Amy D Guertin, Melissa M Martin, Brian Roberts, Melissa Hurd, Xianlu Qu, Nathan R Miselis, Yaping Liu, Jing Li, Igor Feldman, Yair Benita, Andrew Bloecher, Carlo Toniatti, Stuart D Shumway

**Affiliations:** 1 Department of Oncology, Merck Research Laboratories, 33 Avenue Louis Pasteur, Boston, MA 02115, USA; 2 Amgen, One Amgen Center Drive, Thousand Oaks, CA, 91320, USA; 3 Hudson Alpha Institute, 2117 Shades Crest Road SE, Huntsville, AL, 35801, USA; 4 Department of In Vivo Pharmacology, Merck Research Laboratories, 33 Avenue Louis Pasteur, Boston, MA, 02115, USA; 5 Molecular Biomarkers, Merck Research Laboratories, 33 Avenue Louis Pasteur, Boston, MA, 02115, USA; 6 Screening and Protein Sciences, Merck Research Laboratories, North Wales, 19454, USA; 7 Informatics and Analysis, Merck Research Laboratories, 33 Avenue Louis Pasteur, Boston, MA, 02115, USA; 8 Institute for Applied Cancer Science, 1901 East Rd., Unit 1956 Room 4SCR6.1009, Houston, TX, 77054, USA

## Abstract

**Background:**

Inhibition of kinases involved in the DNA damage response sensitizes cells to genotoxic agents by abrogating checkpoint-induced cell cycle arrest. CHK1 and WEE1 act in a pathway upstream of CDK1 to inhibit cell cycle progression in response to damaged DNA. Therapeutic targeting of either CHK1 or WEE1, in combination with chemotherapy, is under clinical evaluation. These studies examine the overlap and potential for synergy when CHK1 and WEE1 are inhibited in cancer cell models.

**Methods:**

Small molecules MK-8776 and MK-1775 were used to selectively and potently inhibit CHK1 and WEE1, respectively.

**Results:**

In vitro, the combination of MK-8776 and MK-1775 induces up to 50-fold more DNA damage than either MK-8776 or MK-1775 alone at a fixed concentration. This requires aberrant cyclin-dependent kinase activity but does not appear to be dependent on p53 status alone. Furthermore, DNA damage takes place primarily in S-phase cells, implying disrupted DNA replication. When dosed together, the combination of MK-8776 and MK-1775 induced more intense and more durable DNA damage as well as anti-tumor efficacy than either MK-8776 or MK-1775 dosed alone. DNA damage induced by the combination was detected in up to 40% of cells in a treated xenograft tumor model.

**Conclusions:**

These results highlight the roles of WEE1 and CHK1 in maintaining genomic integrity. Importantly, the strong synergy observed upon inhibition of both kinases suggests unique yet complimentary anti-tumor effects of WEE1 and CHK1 inhibition. This demonstration of DNA double strand breaks in the absence of a DNA damaging chemotherapeutic provides preclinical rationale for combining WEE1 and CHK1 inhibitors as a cancer treatment regimen.

## Background

Small molecule inhibitors against checkpoint kinases constitute a promising class of targeted cancer therapeutics and many are currently under preclinical or even clinical evaluation. The role of checkpoint kinases is to respond to stress, typically damaged DNA or aberrant chromosomal structure, and stop the cell division process long enough for the damage to be repaired. These “checkpoints” prevent cells from dividing and perpetuating mutations or chromosomal anomalies that would otherwise lead to cellular lethality. The rationale for inhibiting checkpoint kinases is to accumulate irreparable and fatal genetic lesions by compromising the DNA damage response (DDR) and forcing premature or untimely cell division. Notable examples include the mitotic checkpoint kinases Aurora A and B, checkpoint kinase 1 (CHK1), CHK2, ATR, and WEE1.

Several CHK1 inhibitors have been employed in early stage clinical trials [1,2]. Notably, MK-8776 (also referred to as SCH-900776), a CHK1-selective inhibitor, is under evaluation in phase I studies in combination with gemcitabine or cytarabine [3]. Only one inhibitor of WEE1 has been explored clinically. MK-1775, a potent and selective inhibitor of WEE1, achieved favorable phase I pharmacokinetic and pharmacodynamic endpoints in combination with carboplatin, cisplatin, and gemcitabine, and is under further investigation as a chemosensitizer in a phase II trial [4].

CHK1 is an essential serine/threonine kinase involved in S- and G2/M-phase checkpoints [5-9], replication initiation and fork stability [10-12], homologous recombination repair [13,14], and entry into mitosis in normal cycling cells [15]. Importantly, CHK1 is necessary for unperturbed DNA replication and cell cycle coordination even in the absence of any exogenous insult [16]. The cytotoxic nature of CHK1 knockdown or inhibition, either alone or in combination with DNA-damaging therapeutics, has been described extensively (for review, see [2]).

WEE1 is an essential tyrosine kinase that is also involved in S and G2/M checkpoints. WEE1 directly phosphorylates and inhibits CDK1 and CDK2 at the conserved tyrosine 15 residue, affecting entry into mitosis as well as coordination of DNA replication events. WEE1 is therefore critical for properly timing cell division in unperturbed cells, and loss of WEE1 results in chromosomal aneuploidy and accumulated DNA damage [17]. Additionally, WEE1 is critical to S- and G2/M-phase checkpoint responses following DNA damage as well as in unperturbed cells [18-20]. Interfering with WEE1 has been shown to repress cancer cell proliferation and sensitize theme to the anti-tumor growth effects of DNA-damaging chemotherapeutics or radiation therapy [21-28].

Considering the overlapping roles of WEE1 and CHK1 in mitotic entry, DNA replication, and the DDR, we sought to determine whether inhibition of these two kinases was redundant or complimentary. We demonstrate here that combination of a CHK1 inhibitor, MK-8776, and a WEE1 inhibitor, MK-1775, results in synergistic inhibition of cell proliferation in several human tumor cell lines. Minimal concentrations of the drugs required to block cell proliferation lead to a greater than additive increase of γH2AX, a marker of DNA double strand breaks (DSB). This occurs primarily in S-phase cells, suggesting that the unique combination of CHK1 and WEE1 inhibitors disrupts DNA replication and its associated checkpoint. Pharmacodynamic (PD) analysis in xenograft tumors supports this notion, showing an increase in both the percentage of cells containing DNA damage as well as the duration of the DDR. Consistent with the PD data, we demonstrate that the combination of CHK1 and WEE1 inhibitors leads to greater-than-additive tumor growth inhibition in two human tumor xenograft models. Collectively, these data demonstrate the synergistic anti-tumor effects of pharmacological WEE1 and CHK1 inhibition and highlight the potential of this unique combination in treating human cancer independently of chemotherapeutic drugs.

## Results and discussion

### Inhibition of WEE1 and CHK1 causes synergistic inhibition of cell proliferation

**Figure 1:**
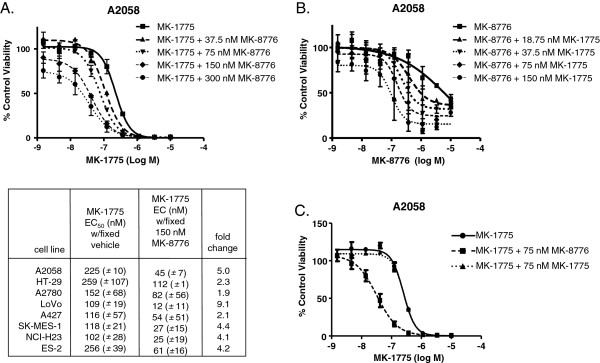
**Restraint of cell proliferation by WEE1 inhibitor is potentiated by a CHK inhibitor, and vice versa.A**, The WEE1 inhibitor MK-1775 was titrated over A2058 cells in addition to either vehicle (DMSO), or the indicated fixed concentration of the CHK1 inhibitor, MK-8776. Tabulated EC_50_ concentrations for MK-1775 in the presence of vehicle or 150 nM fixed concentration of MK-8776 are presented in the lower panel for seven different cell lines. Fold change in EC_50_ values between vehicle treated and MK-8776 treated cells illustrates the potentiation of MK-1775 by MK-8776. **B**, Experiment was performed on A2058 cells as described in (A) with the exception that MK-8776 was titrated over either vehicle (DMSO) or fixed concentrations of MK-1775. **C**, As described in (A), A2058 cells were treated with a gradient of MK-1775 in the added presence of vehicle or 150 nM fixed concentration of MK-1775 or MK-8776.

In a drug combination screen of 39 cell lines, the pairing of MK-1775 (WEE1 inhibitor) and MK-8776 (CHK1 inhibitor) demonstrated synergistic inhibition of proliferation across the majority of cell lines (Additional file 1 Figure S1). To further validate the ability of the two drugs to potentiate the activity of one another, we performed 9-point titrations of each in the added presence of increasing, but fixed, concentrations of the complimentary drug in eight cell lines (Figure 1A, B). In the A2058 melanoma cancer cell line, MK-1775 caused complete growth inhibition with an average EC_50_ of 225 nM. The addition of MK-8776 at concentrations that by themselves do not affect A2058 proliferation (37.5 or 75 nM) caused a shift of the MK-1775 response curve, effectively lowering the EC_50_ of MK-1775 (Figure 1A). Addition of 150 nM MK-8776 reduced the MK-1775 EC_50_ by 5-fold to an average of 45 nM. EC_50_ shifts in other cell lines fell between 1.9- and 9.1-fold (Figure 1A and data not shown). When the converse experiment was performed and MK-8776 was titrated over a range of fixed amounts of MK-1775 in A2058 cells, we again observed leftward shifts in EC_50_ curves as well as a dose-dependent increase in the maximum cell growth inhibition attained at the highest concentration of MK-8776 (Figure 1B). Synergy values for MK-1775 and MK-8776 were notably low in two primary cell lines examined, human mammary epithelial cells and human renal epithelial cells (Additional file 1 Figure S1 and Additional file 2 Figure S2).

Inhibition of WEE1 and CHK1 leads to aberrant CDK1 and/or CDK2 activity, the possible mechanism underlying the deleterious effects on actively dividing tumor cells. Because of the possible overlap in MK-1775 and MK-8776 mechanisms of action, we carried out sham synergy experiments. We titrated MK-1775 over 75 nM of MK-1775 itself or the CHK1 inhibitor MK-8776 and confirmed that MK-1775 did not cause its response curve to shift whereas MK-8776 caused a robust potency shift (Figure 1C). These findings highlight the complimentary, non-overlapping mechanisms underlying the in vitro synergy of WEE1 and CHK1 inhibitors.

### Cell cycle disruption results from WEE1 and CHK1 inhibition

**Figure 2:**
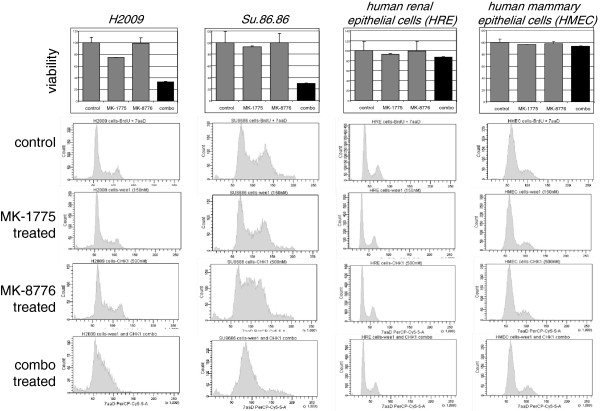
**MK-1775 combined with MK-8776 results in G1/S-phase accumulation.** Cancer cell lines or primary human epithelial cells were treated with vehicle control (DMSO), 150 nM MK-1775, 500 nM MK-8776, or both compounds for 72 hours and analyzed for cell viability (top panels). Values are normalized and graphed as viable percentage of treated cells relative to DMSO treated control cells. Bottom panels, cells were treated with the same concentrations of MK-1775 and MK-8776 used in the upper panels but only for 24 hours before being harvested and analyzed by flow cytometry for DNA content.

As negative regulators of CDKs, both WEE1 and CHK1 coordinate passage through the cell cycle. Therefore, we determined the effects of simultaneous inhibition of WEE1 and CHK1 on the cell cycle profile of asynchronously growing cell populations. We selected two cell lines where synergy was observed, NCI-H2009 NSCLC and Su.86.86 pancreatic cancer cells, and used concentrations of the two inhibitors that had little effect on cell proliferation when dosed alone, but had a profound effect in combination. We also included normal human renal epithelial (HRE) cells and normal human mammary epithelial cells (HMEC), which did not score high in the combination synergy screen (Additional file 1 Figure S1) and were unresponsive to the concentrations of inhibitors used (Figure 2, top panel, 150 nM MK-1775 and 500 nM MK-8776). In all the cells tested, MK-1775 had no discernible effect on the cell cycle profile, whereas MK-8776 caused an increase in the number of G1/S-phase cells in the cancer lines (Figure 2, bottom panels). Notably, the combination of MK-1775 and MK-8776 led to a dramatic accumulation of cells with G1/S-phase DNA content in both tumor lines (Figure 2, bottom row). Again, this effect was not seen in either of the normal cell populations tested, consistent with published observations that WEE1 knockdown is more deleterious in transformed cells than in non-transformed cells [27].

### Inhibition of WEE1 and CHK1 leads to synergistic accumulation of DNA damage

**Figure 3:**
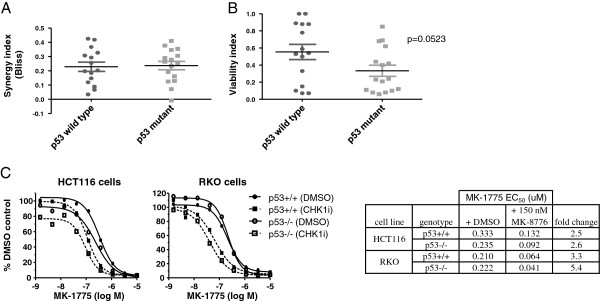
**Cellular p53 status does not predict synergy when MK-1775 is combined with MK-8776.A**, Proliferation assays were performed on cell lines treated with four concentrations each of MK-1775 and MK-8776 titrated against each other (see Additional file 1: Figure S1). Synergy, calculated as the volumetric difference between the observed response and the predicted additive response (Bliss), was plotted for p53 wild type cells (n=15) and cell lines harboring p53 mutations (n=16). **B**, As described in part A, except that fractional viability relative to DMSO treated control cells from the proliferation assay was plotted for cell lines treated with 150 nM MK-1775 and 350 nM MK-8776. **C**, Parental and p53-deleted HCT116 and RKO matched pair cells were treated in a proliferation assay with MK-1775 in the presence of either added vehicle (DMSO) or 150 nM MK-8776. Results from the response curves were tabulated and synergy determined by fold change in MK-1775 EC_50_ values.

Inactivating mutations of p53 can impair the G1 checkpoint arrest that typically follows DNA damage. Due to a compromised G1 checkpoint, it is suggested that p53-deficient tumor cells are more dependent on the G2 checkpoint and therefore more likely to be sensitized to DNA damaging agents by G2 checkpoint modulators, i.e. WEE1 or CHK1 inhibitors. Mutational status of p53 was found for 31 of the 39 cancer cell lines screened and these lines were analyzed for synergy of the MK-1775 and MK-8776 combination (Figure 3A). Synergy scores ranged from 0 (no synergy) to > 0.4 (strong synergy) among both p53 wild type and p53 mutant cell lines, but we failed to observed any difference in synergy among the two groups. The same cell panel did demonstrate a trend toward greater overall sensitivity to the MK-1775 plus MK-8776 combination among p53 mutant lines (Figure 3B). Two p53 wild type or null isogenic cell line pairs demonstrated similar synergy and overall response to the MK-1775 and MK-8776 combination (Figure 3C).

**Figure 4:**
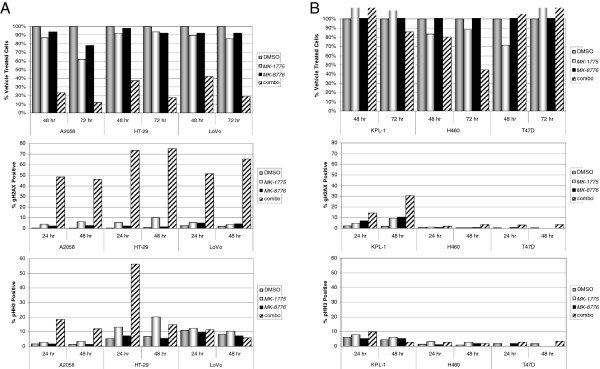
**Combined WEE1 and CHK1 inhibition at anti-proliferative concentrations leads to synergistic induction of DNA damage.A**, Concentrations of MK-1775 and MK-8776 were selected that alone have minimal effects on cell viability but in combination lead to inhibition of cell proliferation. Concentrations used in each cell line were, for A2058: 125 nM MK-1775, 150 nM MK-8776; for HT-29: 125 nM MK-1775, 300 nM MK-8776; and for LoVo: 40 nM MK-1775, 75 nM MK-8776. Top panel, Cells were treated with DMSO, MK-1775, MK-8776, or both compounds and viability for A2058, LoVo, and HT-29 cells was determined at 48 and 72 hours. Viability is shown as percentage of DMSO treated control cells. Middle panel, Cells were treated as above and collected at 24 and 48 hours following drug addition. To assess DNA damage, the percentage of γH2AX positive cells was determined by flow cytometry. Lower panel, Cells were treated as above and collected at 24 and 48 hours for mitotic index analysis, calculated by determining the percentage pHH3 positive cells by flow cytometry. **B**, KPL-1, NCI-H460, and T47D cell lines were treated with DMSO, 150 nM MK-1775, 300 nM MK-8776, or the combination of the two drugs and analyzed for viability, DNA damage, and mitotic index as described in (A).

Loss of either WEE1 or CHK1 function through siRNA depletion or small molecule inhibition results in an accumulation of DNA damage. Therefore, we considered the likelihood that combining MK-1775 with MK-8776 might result in increased DNA damage. To differentiate effects of the combination from effects of either single agent, we selected concentrations of MK-1775 and MK-8776 that alone had limited effect in a cell proliferation assay, but when combined led to >80% growth inhibition (Figure 4A, top panel). We selected three sensitive cell lines where the combination of MK-1775 and MK-8776 was effective: A2058 (p53 mutant) melanoma cells treated with 125 nM MK-1775 and 150 nM MK-8776, HT-29 (p53 mutant) colorectal cancer cells treated with 125 nM MK-1775 and 300 nM MK-8776, and LoVo (p53 wild type) colorectal cancer cells treated with 40 nM MK-1775 and 75 nM MK-8776. Continuous exposure to either drug individually for as long as 48 hours was unable to robustly induce γH2AX staining, appearing in only 10% or less of treated cells (Figure 4A, middle panel). When combined, however, the same concentrations of MK-1775 and MK-8776 demonstrated synergistic induction of γH2AX in as many as 45% to 75% of treated cells, which was maximally induced by 24 hours.

In addition to their effects on DNA metabolism, both WEE1 and CHK1 inhibitors are known to disrupt the G2/M cell cycle checkpoint and accelerate mitotic entry. Therefore, to determine whether premature mitosis could contribute to the synergism of MK-1775 and MK-8776, the mitotic index of treated cells was scored using the mitotic marker phosphorylated serine 28 of histone H3 (pHH3). In both HT-29 and, to a lesser extent, A2058 cells, we observed a more-than-additive increase of pHH3 positive cells in the combination treated population, indicating that accelerated or premature mitosis can indeed result from combination treatment (Figure 4A, lower panel). Interestingly, however, no increase in pHH3 positive cells was observed when LoVo cells were treated with the combination despite equally robust inhibition of cell proliferation and induction of DNA damage as in the A2058 and HT-29 cells. This suggests that DNA damage is the primary mechanism underlying the cytotoxic synergy of WEE1 and CHK1 inhibitors, whereas premature mitosis may or may not contribute as a secondary mechanism of action.

To better determine whether DNA damage is associated with the anti-proliferative effect of the drug combination, we analyzed three less responsive cell lines for induction of γH2AX or pHH3 (Figure 4B). We treated KPL-1 (p53 wild type), NCI-H460 (p53 wild type), and T47D (p53 mutant) cells each with 150 nM MK-1775, 300 nM MK-8776, or both. These drug concentrations are equal to or in excess of those used for the three sensitive cell lines. As expected, only minimal effects were observed on cell viability. In KPL-1 cells we observed induction of γH2AX, though unlike all three sensitive lines, the DNA damage in the combination treated sample was not maximally induced by 24 hours, did not exceed 32%, and was not obviously supra-additive. This cell line did not show an increase in pHH3 positive cells when treated with the combination. Neither NCI-H460 nor T47D cells showed any appreciable evidence of DNA damage or premature mitosis, supporting the notion that MK-8776 and MK-1775 synergize to inhibit cell proliferation by inducing DNA damage in sensitive cell lines.

### DNA damage is present in the S-phase population of cells and is CDK dependent

**Figure 5:**
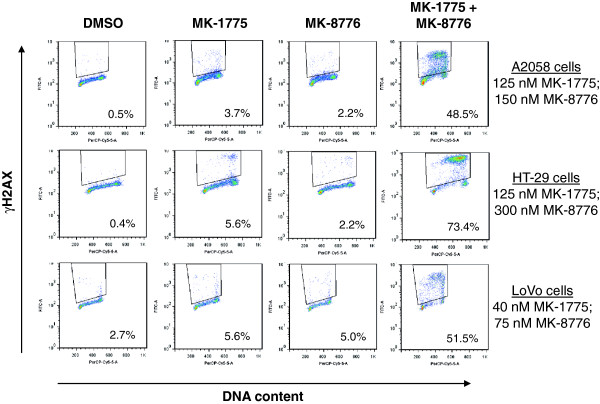
**DNA damage induced by MK-1775 and MK-8776 is present in S-phase cells.** A2058, HT-29, and LoVo cells were treated for 24 hours as in Figure 4 with DMSO or concentrations of MK-1775 and/or MK-8776 indicated. Cells were stained for DNA content (x-axis) and γH2AX (y-axis). γH2AX-positive cells are outlined and percentage of γH2AX-positive cells is indicated.

**Figure 6:**
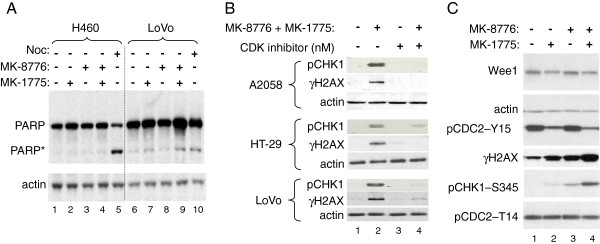
**DNA damage response incurred by MK-1775 and MK-8776 is dependent on CDK activity.A**, Resistant (H460) or sensitive (LoVo) cells were treated with concentrations of MK-1775 and MK-8776 described for Figure 4, or 1 uM nocodazole for control. After 24 hours, cells were harvested and lysates analyzed by Western blot for caspase-dependent cleaved PARP (PARP*). **B**, A2058, HT-29, and LoVo cells were treated for 30 minutes with either DMSO or the indicated concentration of CDK inhibitor (SCH-727965). Following this pretreatment, further DMSO or concentrations of MK-1775 and MK-8776 used in Figures 3 and 4 (125 nM MK-1775 plus 150 nM MK-8776 in A2058; 125 nM MK-1775 plus 300 nM MK-8776 in HT-29, and 40 nM MK-1775 plus 75 nM MK-8776 in LoVo) were added to the cells for an additional 2 hours before cells were harvested and lysates analyzed by Western blot for phosphorylated CHK1^S345^, indicative of activated DNA damage response. **C**, LoVo cells were treated for 2 hours with 75 nM MK-1775 alone or in combination with 150 nM MK-8776, as indicated. Cells were harvested and lysates analyzed by Western blot for the proteins and phosphoproteins indicated.

Both siRNA knockdown and pharmacologic inhibition of WEE1 are known to result in damaged DNA specifically in S-phase cells ( [19], data not shown). Cell cycle analysis based on DNA content of the three sensitive cell lines above demonstrated that DNA damage caused by the MK-1775 and MK-8776 combination is detected in S- phase (Figure 5). Detection of cleaved PARP in the presence of the drug combination suggests apoptosis as a result of DNA damage in sensitive cell lines (Figure 6A).

Since the only characterized substrates for WEE1 are CDK1 and CDK2, we next questioned whether the ability of WEE1 and CHK1 inhibition to result in DNA damage was dependent on CDK activity. For these studies we employed SCH-727965, a previously described potent inhibitor of CDK1, CDK2, CDK5, and CDK9 [29]. We looked at phosphorylated serine 345 of CHK1 in the three sensitive cell lines as a surrogate for an activated DNA damage response [9]. As expected, pairing of MK-1775 and MK-8776 at concentrations that induced γH2AX (Figure 5) also led to the rapid phosphorylation of CHK1^S345^ and induction of γH2AX (Figure 6B). Notably, this phospho-CHK1^S345^ signal was reduced by a 30 minute pretreatment of the CDK inhibitor, implying that aberrant CDK activity, as a result of WEE1 and/or CHK1 inhibition, is required for the drug combination to induce DNA damage. Although we monitored acute induction of DNA damage, we cannot exclude the possibility that CDK inhibition arrests cell cycle progression, indirectly preventing DNA damage following MK-1775 and MK-8776 treatment.

To ask whether MK-1775 and MK-8776 act cooperatively to increase CDK activity through reduced inhibitory phosphorylation, we determined the phosphorylation status at CDK1^T14^ and CDK1^Y15^ in LoVo cells treated alone or in combination. As Figure 6C shows, treatment with MK-1775 resulted in an expected decrease of phospho-CDK1^Y15^, no detectable change in phospho-CDK1^T14^, and slight induction of the DDR evident from phospho-CHK1^S345^. Treatment with MK-8776 also induced the phospho-CHK1^S345^ signal, which was even further increased following treatment with the combination. Interestingly, however, neither MK-8776 nor MK-8776 in combination with MK-1775 led to further reduction of phospho-CDK1^Y15^, suggesting that MK-8776 might be cooperating with MK-1775 via modulation of downstream effectors of CHK1 other than CDC25 phosphatases and CDKs. This finding is consistent with the observed synergy of MK-1775 and MK-8776 and the notion that WEE1 and CHK1 carry out unique, yet complimentary, functions in DNA replication and/or intra-S phase checkpoint control.

### MK-1775 and MK-8776 lead to increased DNA damage in xenograft models

**Figure 7:**
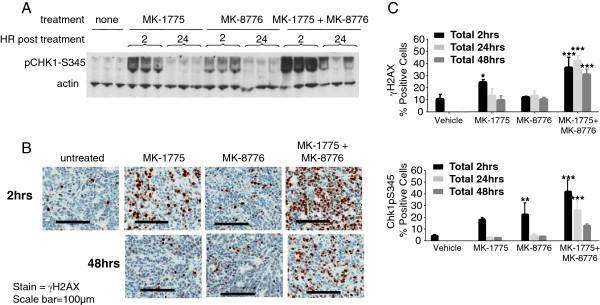
**Cooperative induction of DNA damage in vivo by WEE1 and CHK1 inhibitors.** LoVo xenograft tumor-bearing mice were treated with 60 mpk MK-1775 BID for 2 days, 60 mpk MK-8776 BID for 2 days, or the combination of MK-1775 and MK-8776 each at 60 mpk BID for 2 days. Tumors were collected at 2, 24, and 48 hours following the final dose. **A**, LoVo tumor lysates were analyzed by Western blot for pCHK1^S345^. **B**, Tumor sections were fixed and analyzed by immunohistochemistry (IHC). Representative images for γH2AX at 2 hours and 48 hours post final dose are shown. **C**, Quantitative analysis of IHC for both phospho-CHK1^S345^ and γH2AX (n=3); one-way ANOVA analyses *P<0.05. **P<0.01, ***P<0.001.

Combination of MK-1775 and MK-8776 synergistically induced DNA damage in vitro (Figure 4), so we next examined its effect on DNA damage in vivo. Animals bearing LoVo xenograft tumors received 2 days of twice daily (BID) dosing of vehicle, MK-1775 (60 mpk), MK-8776 (60 mpk), or the combination. Tumors were collected at 2, 24, and 48 hours after the fourth and final dose and subsequently analyzed by Western blot and immunohistochemistry (IHC). Figure 7A shows that when dosed alone, both MK-1775 and MK-8776 lead to a transient increase in phospho-CHK1^S345^. Notably, treatment with the combination resulted in a greater induction of phospho-CHK1^S345^ at 2 hours, and unlike either single agent alone, this effect was still evident at 24 hours after the final dose. Consistent with this observation, IHC results in Figure 7B demonstrate an increase in the intensity and duration of the γH2AX signal when the combination is used relative to either single agent. Quantitation of the IHC results (Figure 7C) shows that this is true of both the γH2AX as well as the phospho-CHK1^S345^ DNA damage signals.

### In vivo xenograft efficacy from WEE1 and CHK1 inhibition

**Figure 8:**
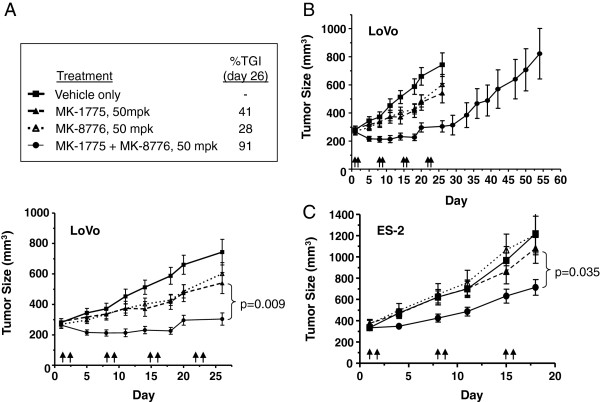
**Activity of MK-8776 (i.p.) and MK-1775 (p.o.) in LoVo xenograft bearing mice.A**, Each compound, alone or in combination, was dosed at 50 mg/kg BID for 2 days per week over 4 weekly cycles and is indicated with arrows at the bottom of the graph. Control and single agent groups received both or one vehicle, respectively, so that all animals were dosed with equal vehicle volume and frequency. Percent TGI was calculated as 100 * ΔT/ΔC if ΔT > 0 where ΔT = final mean volume – initial mean volume of treated group, ΔC = final mean volume – initial mean volume of vehicle control group, and Ti = initial mean volume of treated group. P value was derived by student’s t-test for combination (n=10) versus MK-1775 treated tumors (n=10) on day 26. **B**, In the same study described in (A), LoVo tumor growth was tracked through study day 56 following the fourth and final treatment cycle on days 22 and 23. **C**, ES-2 xenograft tumors were treated as in (A) for 3 treatment cycles, and days of drug administration are indicated by arrows at the bottom of the graph. P value was derived by student's t-test for combination (n=10) versus MK-1775 treated tumors (n=10) on day 18.

Combination of MK-1775 and MK-8776 induces greater-than-additive DNA damage both in vitro (Figure 4) and in vivo (Figure 7). To determine whether the observed DNA damage translates into efficacy, the anti-tumor effect of this combination was assessed in two xenograft models of human cancer. We used LoVo colorectal cancer cells (Figures 8A and 8B) and ES-2 ovarian carcinoma cells (Figure 8C). Tumor-bearing animals received 2 day BID dosing of (i) vehicle only, (ii) MK-1775 (50 mpk) plus vehicle, (iii) MK-8776 (50 mpk) plus vehicle, or (iv) MK-1775 (50 mpk) plus MK-8776 (50 mpk).

Treatment with either MK-8776 or MK-1775 alone had a modest effect on growth in LoVo xenografts, resulting in 28% or 41% tumor growth inhibition (TGI), respectively (Figure 8A). Based on the single agent treatment arms, the Bliss independent (BI) model predicts 58% TGI for additive effects of combination (see Methods). However, at the same doses and schedule used for each single agent, the combination of MK-1775 and MK-8776 resulted in 91% TGI. However, these LoVo xenograft tumors resumed growth shortly after drug treatment was stopped (Figure 8B). In the ES-2 xenograft study, the same treatment schedules of MK-8776 and MK-1775 resulted in 1% and 16% TGI, respectively. We observed 57% TGI in the combination treatment arm, which was a notable 40% above the 17% TGI predicted for the combination by the BI model if the two drugs acted additively. Mean body weight loss for the combination treatment group in either study did not exceed 8%, and even then only for initial and not subsequent doses, indicating that efficacy was achieved at tolerated drug combination exposures (data not shown). These data support the notion that combined inhibition of WEE1 and CHK1 achieves in vivo synergy and highlights the potential of this unique drug combination in the treatment of human neoplasms.

## Conclusions

Using small molecule inhibitors currently under early clinical development, we have shown that simultaneous inhibition of the WEE1 and CHK1 kinases results in synergistic potentiation of each drug for a variety of cell types in proliferation assays. Knockout of WEE1 results in embryonic lethality before day 3.5 [17], and knockdown of WEE1 is known to inhibit proliferation of several cancer cell lines in vitro [27,28]. Similarly, anti-proliferative effects of CHK1 inhibition via siRNA or pharmacologic inhibition have been described [30]. The increased potency of MK-1775 and MK-8776 when combined supports the notion that WEE1 and CHK1 have non-overlapping activity. Potentially predictive biomarkers for each class of drug have been described for their chemosensitizing effects, including p53 status for both WEE1 and CHK1 [2,31], WEE1 expression levels for WEE1 [28], and cyclin B levels for CHK1 [32]. Interestingly, synergy between MK-1775 and MK-8776 did not correlate with the p53 status of the cell line, though overall sensitivity to the drugs might favor p53 mutant lines. Furthermore, three of the seven lines described in Figure 1 are wild type for p53 (A2780, LoVo, and A427). Further examination of other putative markers such as expression of WEE1, CHK1, or cyclin B1, will be important future questions to address in understanding the cellular context of WEE1 and CHK1 inhibitor activity.

Mechanistic studies suggest that WEE1 and CHK1 inhibitors combine synergistically due to, at least in part, alterations of the cell cycle and compounded DNA damage (Figures 2, 3, and 4). Though both MK-1775 and MK-8776 are chemosensitizers that potentiate the anti-proliferative effects of DNA damaging chemotherapeutics, it is also known that knockdown or inhibition of either WEE1 or CHK1 alone leads to DNA damage. Therefore, it is likely that MK-1775 and MK-8776 work together in an analogous fashion as they do in combination with genotoxic agents to prevent proper checkpoint response and damage control. Importantly, DNA damage incurred by WEE1 and CHK1 inhibition occurs primarily in S phase and requires CDK activity, consistent with findings that disruption of either WEE1 or CHK1 individually leads to S-phase arrest, slowed DNA replication, and induced DNA damage. Increased accumulation and duration of DNA damage by MK-1775 and MK-8776 was observed in vivo, and accordingly the combination led to inhibition of tumor growth in xenograft models. WEE1 and CHK1 inhibition was unable to prevent tumor regrowth, however, suggesting either that not all cells are affected or that following drug treatment cells are able to sufficiently repair damaged DNA. Along these lines, we were unable to find robust evidence of apoptosis both in vitro and in vivo (data not shown).

The WEE1 inhibitor MK-1775 is known to reduce phosphorylation on tyrosine 15 of CDK1/2, resulting in increased CDK1/2 activity [26]. Inhibition of CHK1 increases the activity of the protein phosphatases CDC25A/B/C, thereby reducing phosphorylation of tyrosine 15 and indirectly increasing CDK1/2 activity. We hypothesized, therefore, that combined inhibition of WEE1 and CHK1 could result in an additive inhibition of phospho-CDK1/2^Y15^. However, we were unable to observe a substantial decrease in phospho-CDK1/2^Y15^ beyond the effect of MK-1775 alone, suggesting that CHK1 inhibition by MK-8776 compliments inhibition of WEE1 through mechanism(s) and target(s) distinct from CDK1/2.

The synergistic antiproliferative effect of combined WEE1 and CHK1 inhibition was also noted by Davies et al. [33] and Carrassa L et al. [34]. Each of these studies identified the WEE1 gene as an siRNA target that could sensitize to either a CHK1 inhibitor (Davies et al.) or a CHK1 siRNA (Carrassa et al.) in solid tumor cell lines. Davies et al. reported synergy between WEE1 and CHK1 inhibitors in four cell lines, three of which are reported p53 wild type [33]. Similarly, Carrassa et al. reported synergy in seven cell lines regardless of p53 status [34]. This manuscript extends earlier findings into 37 cancer cell lines using compounds that are currently under early stage clinical development. Our findings align with those reported demonstrating that the mechanism underlying synergy between WEE1 and CHK1 inhibition is ubiquitous as well as with the finding that p53 status does not affect this synergy.

Davies et al. reported an absence of premature mitosis in the HEL92.1.7 cell line, though this experiment was conducted with an excess of WEE1 and CHK1 inhibitors required for inhibition of cell proliferation (compare Figures 2C and 5 in [33]). Carrassa et al. conducted mechanistic studies in one cell line, OVCAR-5, and concluded that premature mitosis accompanied the simultaneous inhibition of WEE1 and CHK1 inhibition [34]. It was unclear in that study whether concentrations of inhibitors used to study biochemical correlates coincided with the concentrations required to inhibit proliferation. By examining the effects of MK-1775 and MK-8776 at the lowest concentrations needed to achieve antiproliferative activity, individualized for multiple cell lines, we are able to demonstrate that DNA damage rather than premature mitosis seems to be the primary cause of synergistic cytoxicity (Figure 4), though we do find that select cell lines, i.e. HT-29, may undergo premature mitosis as well. Importantly, these findings were corroborated in vivo where LoVo xenograft tumor samples demonstrated synergistic increases in the DNA damage markers γH2AX and pCHK1^S345^ but not in the mitosis marker pHH3 (Figure 7 and data not shown). Collectively these data argue that nonoverlapping functions of the WEE1 and CHK1 kinases during S- phase are responsible for the widespread and strong synergy observed following their inhibition.

Our studies describe synergy achieved by simultaneous inhibition of the WEE1 and CHK1 kinases and, together with the work of Davies et al. [33] and Carrassa et al. [34], provide pharmacologic evidence that the two kinases have unique and nonoverlapping activities. Combined treatment with MK-1775 and MK-8776 demonstrates synergistic DNA damage and anti-tumor efficacy at tolerated doses, suggesting possible clinical use of the drugs in combination. The robust and ubiquitous nature of the synergy may suggest potential toxicity in normal tissue and therefore identification of mechanisms underlying sensitivity will be important in understanding the potential clinical application of this combination.

## Methods

### Cell culture and compounds

All cell lines were obtained from American Type Culture Collection, except A2780 cells which were obtained from Sigma, and cultured under vendors’ recommended conditions. The HCT116 and RKO isogenic cell lines were obtained from Horizon Discovery, LTD. The chemical name of MK-1775 is (2-allyl-1-[6-(1-hydroxy-1-methylethyl) pyridin-2-yl]-6-{[4-(4-methylpiperazin-1-yl) phenyl]amino}-1,2-dihydro-3H-pyrazolo[3,4-d] pyrimidin-3-one), and its chemical structure is described elsewhere [26]. The chemical name of MK-8776 is *(R)-(−)-*6-Bromo-3-(1-methyl-1H-pyrazol-4-yl)-5-piperidin-3-yl-pyrazolo[1,5-a]pyrimidin-7-ylamine and has been described previously as SCH-900776 [35]. SCH-727965 has also been previously described in the literature [29].

### Cell viability assay

For each experiment, cells were seeded in duplicate 96-well white walled plates at 4,000 cells per well. After overnight incubation, cells were treated with combinations of DMSO as vehicle control, MK-1775, and MK-8776 for 72 hours. Cell viability was determined by measuring ATP with Vialight Plus (Lonza) according to manufacturer’s instructions. Drug potency was calculated as the ratio of relative light units (RLUs) in compound treated wells over DMSO-treated control wells and expressed as % DMSO control. Compound EC_50_s were calculated in GraphPad Prism using a 4 parameter variable slope sigmoidal dose response curve fit.

### Flow cytometry

Cells were treated with indicated concentrations of MK-1775, MK-8776, both, or an equivalent volume of vehicle for a fixed time period. At time of harvest, cells were counted and then fixed in ice cold 70% ethanol overnight before staining with anti-phospho-histone H2AX (S139; γH2AX) antibody conjugated to FITC (from Millipore kit 17–344), anti-phospho-histone H3 (S28; pHH3) antibody conjugated to Alexa Fluor® 647 (BD Biosciences 558217), and propidium iodide (BD Biosciences 550825). Samples were read on the LSR II (BD Biosciences 347545) flow cytometer, and data were analyzed using FlowJo software version 7.5.5.

### Animals and xenograft studies

CD-1 Nu/Nu female mice aged 5–6 weeks were obtained from Charles River Laboratories and housed in our animal care facility at standard laboratory conditions and fed 2018S autoclaveable diet and water *ad libitum*. The protocol was approved by Merck’s Institutional Animal Care and Use Committee. Mice were inoculated with 5 x 10^6^ LoVo cells in 100 μL (1:1 Matrigel:PBS) subcutaneously (S.C.) into the right flank. When tumor volume reached 200 mm^3^ (+/−50) mice were pair-matched so each group had a similar mean and standard deviation. Tumor volume and body weights were recorded bi-weekly. Mice received 4 treatment cycles of twice daily dosing (BID) for 2 days receiving either vehicle, MK-1775 (50 mpk), and/or MK-8776 (50 mpk) For pharmacodynamic assays, mice were dosed with 60 mpk of each compound.

### In vivo pharmacodynamic assays

Xenograft tumors were fixed in 10% formalin, paraffin-embedded and sectioned at 5 μm. Tumor sections were immunostained with rabbit monoclonal anti-phospho-CHK1 (S345) antibody (1:300 dilution; Cell Signaling); rabbit polyclonal anti-gamma-histone H2AX antibody (1:2000 dilution; Bethyl); rabbit polyclonal anti-phospho-CDC2 Y15 antibody (1:960; R&D Systems) and rabbit monoclonal anti-Ki67 antibody (1:200; Epitomics). Labeled antigens were visualized using Omni Map anti-rabbit HRP and peroxidase substrate (Ventana Medical Systems). Slides were digitized using an Aperio ScanScope XT Image System and immunostained cells were quantified using Aperio Imagescope software. The percentage of cells showing immunostaining in each tumor was calculated relative to the number of total cells with necrotic regions excluded.

### Bliss synergy calculations

The Bliss independence (BI) model is used to define the effect of two drugs assumed to act through independent mechanisms [36]. BI is described by the equation *E*_*i*_ = (*E*_*A*_ + *E*_*B*_) - (*E*_*A*_ x *E*_*B*_), where *E*_*i*_ is the predicted effect (percentage of inhibition) by the combination of drugs *A* and *B* if they were to act additively and independently, and *E*_*A*_ and *E*_*B*_ are the observed effects (percentage of inhibition) of each drug alone, respectively. When observed inhibition exceeds predicted inhibition, the two compounds are considered to act synergistically.

## Misc

Amy D Guertin and Melissa M Martin contributed equally.

## Competing interests

AG, XQ, NM, YL, JL, IF, YB, AB, and SS are employees of Merck Research Laboratories. The author(s) declare that they have no competing interests.

## Authors’ contributions

AG and MM both participated in proliferation assays, carried out flow cytometry for γH2AX, and performed Western blot analyses of cell line studies. YL and JL performed the combination screen of MK-1775 and MK-8776 on solid tumor cell lines. BR, YB, and IF performed synergy analyses of the screen. MH performed xenograft efficacy studies and xenograft sample collection. XQ and NM conducted and quantitated immunohistochemistry assays on xenograft tumor samples. AB contributed to study design and concept. CT contributed to study design, concept, and manuscript preparation. SS participated in proliferation assays, study design and coordination, figure construction, and drafting of the manuscript. All authors read and approved the final manuscript.

## Supplementary Material

Additional file 1 **Figure S1.** Synergistic interaction of MK-1775 and MK-8776 in 39 solid tumor cell lines. A, Cell lines are grouped according to cancer type. Observed synergy is reported for each line as vBliss, which is the volumetric difference between the surface of predicted combination effect and the surface of observed combination effect as illustrated in parts B and C, (see Methods for explanation of Bliss synergy predictions). B, The A2058 melanoma cell line is an example of synergy. Four concentrations each of MK-1775 and MK-8776 were titrated and proliferation at 96 hours was plotted as a fraction of DMSO treated control A2058 cells. The predicted effect on proliferation (using Bliss synergy model) is represented as the upper surface on the plot whereas the observed effect on proliferation is represented by black dots. Observed effects are connected by vertical lines to the corresponding Bliss predicted effect for those concentrations. C, As in part B but showing the KPL1 cell line as an example of lack of synergy between MK-1775 and MK-8776. Click here for file

Additional file 2 **Figure S2.** Synergistic interaction of MK-1775 and MK-8776 in primary human renal epithelial (HRE) cells. A, Proliferation assay results (72 hours) in HRE cells showing the WEE1 inhibitor MK-1775 titrated in addition to either vehicle (DMSO), or the indicated fixed concentration of the CHK1 inhibitor, MK-8776 (compare to Figure 1). B, Proliferation assay results (72 hours) in HRE cells exposed to 8-point titrations of both MK-1775 (starting 4 μM then 1-to-3 dilutions) and MK-8776 (starting 10 μM then 1-to-3 dilutions) are expressed as surface plots for Bliss predicted additivity and actual observed response (compare to Additional file 1: Figures S1B and S1C). The observed vBliss was 0.06 (compare to Additional file 1: Figure S1A). Click here for file
