# Supplementary material for: Unique functions of CHK1 and WEE1 underlie synergistic anti-tumor activity upon pharmacologic inhibition
Source: Cancer Cell Int. 2012 Nov 13;12:45. doi: 10.1186/1475-2867-12-45 (PMC3517755; doi:10.1186/1475-2867-12-45)
Supplement: Additional file 1 — Figure S1. Synergistic interaction of MK-1775 and MK-8776 in 39 solid tumor cell lines. A, Cell lines are grouped according to cancer type. Observed synergy is reported for each line as vBliss, which is the volumetric difference between the surface of predicted combination effect and the surface of observed combination effect as illustrated in parts B and C, (see Methods for explanation of Bliss synergy predictions). B, The A2058 melanoma cell line is an example of synergy. Four concentrations each of MK-1775 and MK-8776 were titrated and proliferation at 96 hours was plotted as a fraction of DMSO treated control A2058 cells. The predicted effect on proliferation (using Bliss synergy model) is represented as the upper surface on the plot whereas the observed effect on proliferation is represented by black dots. Observed effects are connected by vertical lines to the corresponding Bliss predicted effect for those concentrations. C, As in part B but showing the KPL1 cell line as an example of lack of synergy between MK-1775 and MK-8776. [file 1475-2867-12-45-S1.ppt]

## Slide 1
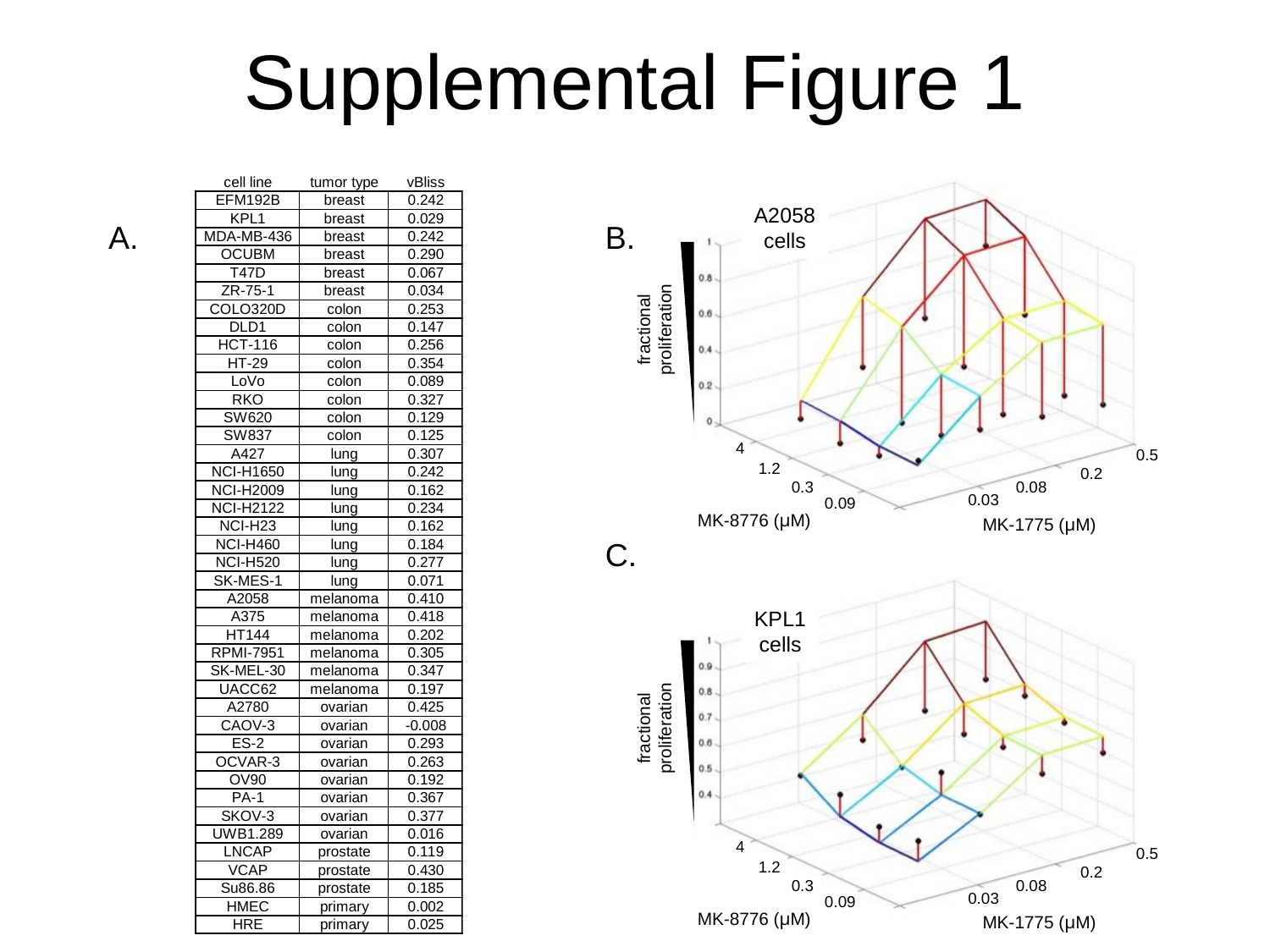

# Supplemental Figure 1
A2058
cells
fractional proliferation
4
0.5
1.2
0.2
0.3
0.08
0.03
0.09
MK-8776 (μM)
MK-1775 (μM)
A.
B.
C.
KPL1
cells
fractional proliferation
4
0.5
1.2
0.2
0.3
0.08
0.03
0.09
MK-8776 (μM)
MK-1775 (μM)
