# Supplementary material for: Unique functions of CHK1 and WEE1 underlie synergistic anti-tumor activity upon pharmacologic inhibition
Source: Cancer Cell Int. 2012 Nov 13;12:45. doi: 10.1186/1475-2867-12-45 (PMC3517755; doi:10.1186/1475-2867-12-45)
Supplement: Additional file 2 — Figure S2. Synergistic interaction of MK-1775 and MK-8776 in primary human renal epithelial (HRE) cells. A, Proliferation assay results (72 hours) in HRE cells showing the WEE1 inhibitor MK-1775 titrated in addition to either vehicle (DMSO), or the indicated fixed concentration of the CHK1 inhibitor, MK-8776 (compare to Figure 1). B, Proliferation assay results (72 hours) in HRE cells exposed to 8-point titrations of both MK-1775 (starting 4 μM then 1-to-3 dilutions) and MK-8776 (starting 10 μM then 1-to-3 dilutions) are expressed as surface plots for Bliss predicted additivity and actual observed response (compare to Additional file 1: Figures S1B and S1C). The observed vBliss was 0.06 (compare to Additional file 1: Figure S1A). [file 1475-2867-12-45-S2.ppt]

## Slide 1
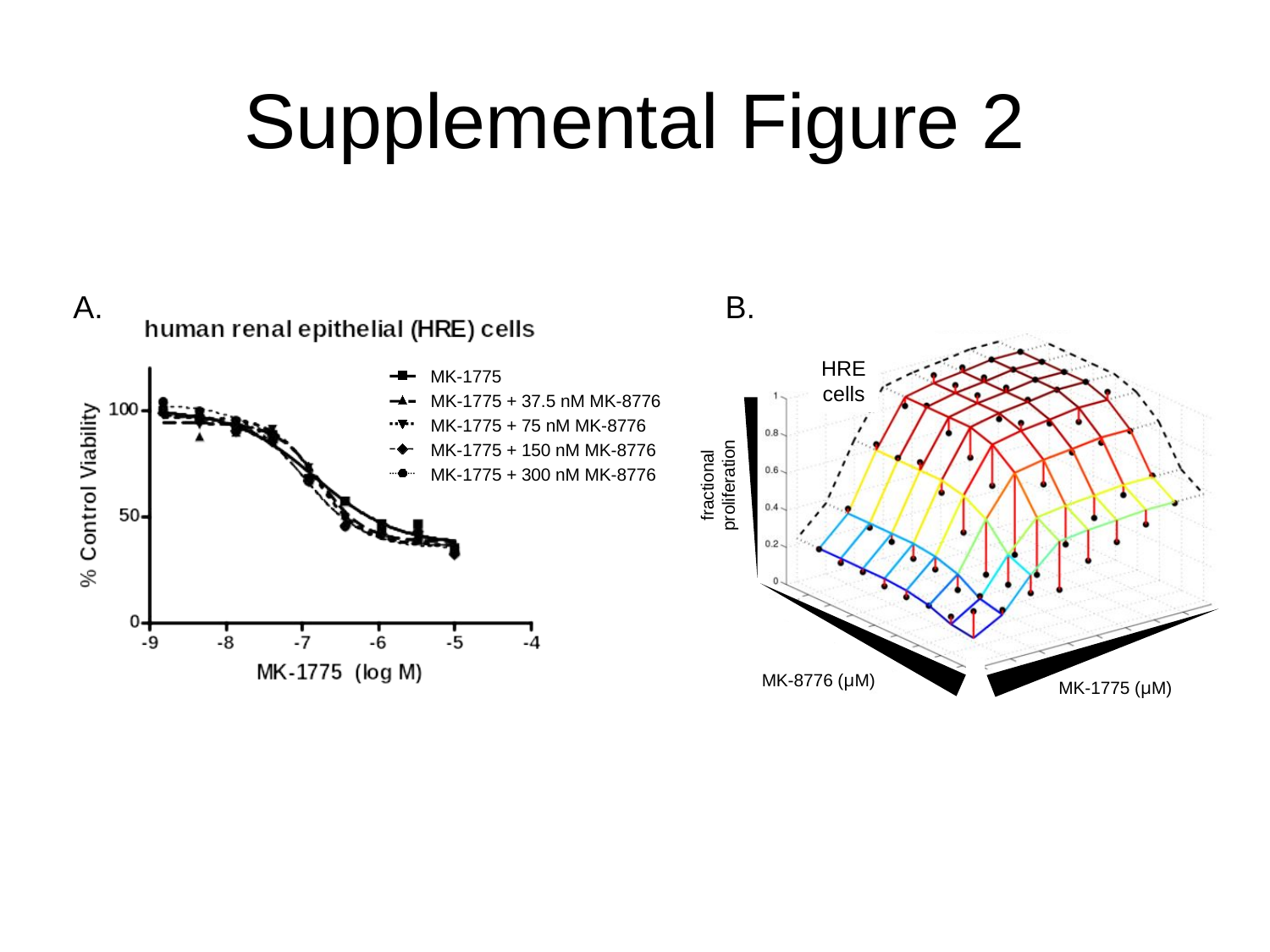

# Supplemental Figure 2
A.
B.
MK-1775
MK-1775 + 37.5 nM MK-8776
MK-1775 + 75 nM MK-8776
MK-1775 + 150 nM MK-8776
MK-1775 + 300 nM MK-8776
HRE
cells
fractional proliferation
MK-8776 (μM)
MK-1775 (μM)
